# Psychiatry in the real world

**DOI:** 10.3389/fpsyt.2025.1616276

**Published:** 2025-07-18

**Authors:** Orestis Giotakos

**Affiliations:** Independent Researcher, Athens, Greece

**Keywords:** psychiatry, mental health, therapy, diagnosis, neuroscience, clinical neuroscience

## Abstract

Although diagnostic and therapeutic issues are relatively settled in formal psychiatry, there is considerable confusion among the public. In the real world, there is notable misunderstanding of terminology and concepts, not only of diagnoses and treatments, but also of the titles and characteristics of mental health professionals and institutions. Moreover, the numerous and conflicting treatment models that have been proposed over time have led to the lack of a clear protocol for the treatment of even a common psychiatric case. Thus, each patient must choose his or her own treatment from among an infinite number of treatment options, provided by an infinite number of therapists, coming from various known and unknown theoretical and therapeutic fields. As a result, psychiatric patients often receive incorrect or incomplete treatments and are faced with chronicity of the disease, exploitation and poverty. Furthermore, the high mental and physical comorbidity of psychiatric cases further exacerbates this situation. We are looking for a psychiatry that will be at the center of the mental health system, having the obligation to constantly regulate the flow of cases, depending on their needs. Psychiatry is grounded in clinical neuroscience. We are looking for psychiatry that will be constantly updated and will apply the valuable knowledge of neuroscience, at a research, diagnostic and therapeutic level. Also, psychiatry that will refer and collaborate closely with other medical specialties, as well as with non-medical accredited mental health professionals. In parallel, psychiatry must envision, plan and supervise the development of scientifically based psychosocial rehabilitation programs. Finally, the stakeholders involved should promote the view of a medico-social approach to mental disorders, centered on formal diagnosis and treatment by professionals, with the aim of providing safe, continuous and complete care for patients.

## The burden of mental illness

What is the real burden of mental illness on the world’s population? How is it changed by phenomena such as pandemics, climate crisis, conflicts or wars? What is the economic cost of these illnesses for states or for the individuals themselves? What percentage of people with mental illnesses access mental health services? What percentage of these people improve or are cured? How much has the progress of (neuro)science contributed to reducing the mental health burden? What is the role of psychiatry and how much can it contribute to the treatment and the wellbeing? Which strategic plan could be the most successful?

The global burden of mental disorders is remarkable both in its impact on human health and losses to societal welfare. About 418 million disability-adjusted life years (DALYs) could be attributable to mental disorders in 2019 (16% of global DALYs), while at a regional level, the losses could account for between 4% of gross domestic product in Eastern sub-Saharan Africa and 8% in High-income North America (1). It is estimated that more than 1 in 5 U.S. adults live with any mental illness, while 1 in 20 U.S. adults (6.0% of all U.S. adults) experience serious mental illness each year. In 2022, among the 59.3 million adults with any mental illness, 30.0 million (50.6%) received mental health treatment in the past year, while 10.2 million (66.7%) received mental health treatment in the past year. Moreover, 1 in 6 U.S. youth aged 6-17 experience a mental health disorder each year, 50% of all lifetime mental illness begins by age 14, and 75% by age 24, while suicide is the 2nd leading cause of death among people aged 10-14 (2). The number of incident cases due to mental disorders in China increased from 42.90 million to 52.72 million, the number of prevalent cases increased from 132.63 million to 160.16 million, and the number of DALYs increased from 15.64 million to 20.29 million, during 1990-2019 (3).

The Global Burden of Disease study assessed the prevalence and burden estimates for 12 mental disorders, males and females, 23 age groups, 204 countries and territories, between 1990 and 2019. It showed that mental disorders remained among the top ten leading causes of burden worldwide, with no evidence of global reduction in the burden since 1990 (4). How can we explain the fact that despite the dizzying increase in technology, neuroscience, therapists and treatments, the number of mentally ill individuals has remained the same for the last 30 years? Are there problems with access to the mental health providing system? Is there any distortion in the service providing process? Are the treatments not ultimately that effective? Are the diagnoses also constructed in the wrong direction? Are there problems of understanding between patients and therapists or even between institutes and services – that is, does everyone speak their own language?

## Timeless misnomers and misconceptions

In the real world, depressed people are not the only ones who have ‘depression’, nor are psychotic people the only ones who have ‘psychosis’, and ‘antidepressants’ are not prescribed only for depression and ‘antipsychotics’ are not prescribed only for ‘psychosis’. The biological basis of mental disorders is poorly understood, while diagnostic criteria tend to change, and diagnostic entities appear or disappear with relative ease in diagnostic manuals. However, in clinical practice it seems that a correct diagnosis is not necessary for patients to receive the best possible treatment. Although the use of explicit “operational criteria” is essential for obtaining reliable clinical assessments, the reliability of DSM criteria does not guarantee that they are the most valid representation of an underlying pathological process (5). Diagnostic terminologies produce various meanings in both patients and clinicians. ‘Depressed mood’ in clinical assessment and questionnaires, is associated with ‘low’ mood, although not a few patients, or even unrecognized patients, suffer from ‘bad’, ‘dysthymic’, ‘negative’ or even ‘empty’ mood. Also, there are many sub-threshold forms of depression, such as a typical depression, characterological depression, neurotic depression, reactive depression, and anxious depression. Do all these patients suffer from depressive disorder or do they have a strong psychological dimension of depression as part of different illnesses?

Moreover, is ‘polarity’ the central parameter in bipolar disorder? ‘Unequivocal’ depression and ‘unequivocal’ mania are rare occurrences, and mixed emotional states are probably the rule. Could the key element then be ‘cyclicality’, or forms of ‘borderline disorder’ or ‘psychosis’? In the case of schizophrenia, due to the connotation of ‘split mind’ and its stigmatizing attributions, the discussion around changing the term of schizophrenia is ongoing (6). Guloksuz and van Os (7), suggested that a name change will reduce stigma and discrimination, although extensive reconceptualization is more challenging than a simple semantic revision. Following the trend in Asian countries (8), various alternatives of the term ‘schizophrenia’ have been proposed by scholars, service patients and professional organizations across the world, each with a different emphasis on conceptualization (9).

**Figure 1:**
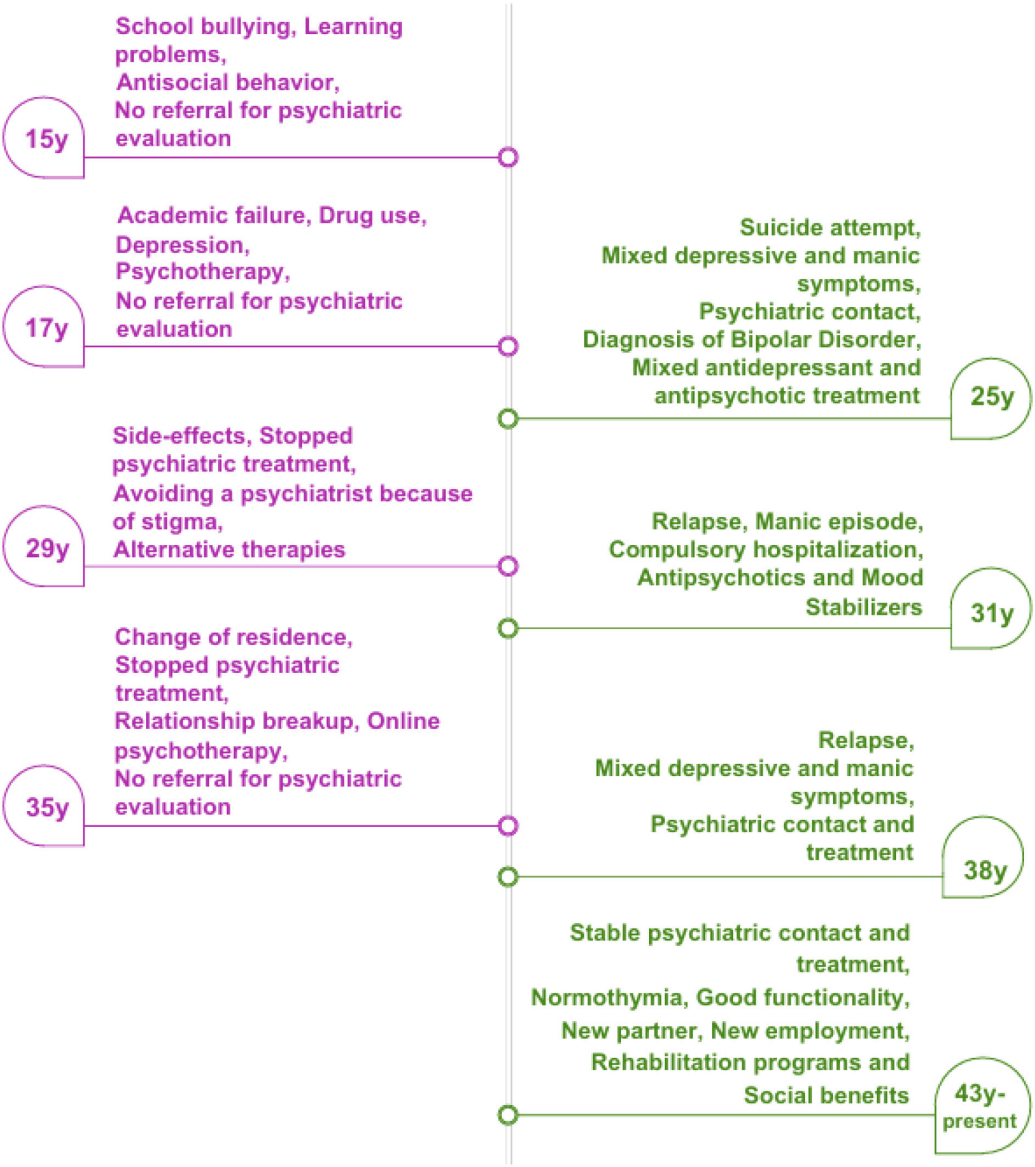
A flowchart of a psychiatric patient, showing the major events during the lack of contact (left side) and the stable contact (right side) with a psychiatrist and psychiatric treatment.

To date, both accurate diagnosis and effective treatment of mental disorders remain unfulfilled goals (10). While the reliability of psychiatric diagnoses has improved significantly with the use of explicit diagnostic criteria, their validity remains uncertain. As a result of diagnostic and therapeutic confusion, psychiatrists need to work as balancers between patients’ needs, treatment guidelines, evidence-based research, meta-analyses, and everyday clinical demands. Over time, the isolation of psychiatry from other medical specialties has diminished the value of diagnosis and treatment, reducing psychiatry to a specialty that provides non-specialized psychological support (11). According to Nancy Andreasen (12), “Validity of psychiatric diagnosis has been sacrificed to achieve reliability. DSM diagnoses have given researchers a common nomenclature - but probably the wrong one. Although creating standardized diagnoses that would facilitate research was a major goal, DSM diagnoses are not useful for research because of their lack of validity”. This diagnostic and therapeutic confusion often leads patients to doubt, lose confidence, choose random or occasional treatments and therapists or even to discontinue. The result of this is the deterioration and relapse of the disease, with devastating consequences for their functionality and socioeconomic status. Often, individuals do not know which mental health specialist they should contact, ultimately choosing with unknown criteria through a random search on the Internet. **Figure 1** depicts the flow of such a case, although one can distinguish elements of this process in almost all cases with a mental illness problem.

The high co-morbidity of almost all mental disorders, with both mental and physical illnesses, coupled with the low to moderate therapeutic response of almost all diagnostic groups to pharmacotherapy and/or psychotherapy, indicate an ambiguity of diagnostic limits and accurate (13). In the case of PTSD, as well as in many other mental disorders, the following paradox is observed: While according to the diagnostic criteria clinicians agree on diagnosis, which means there is a good reliability, it seems as if it is diagnosed something that more or less does not exist, which means validity (both context and face) is very low. PTSD is difficult to be diagnosed reliably due to the high degree of variety of symptoms. An individual should have experienced 1 of the 5 re-experiencing symptoms, 3 of the 7 avoidance symptoms and 2 of the 5 hyperarousal symptoms. Thus, there are hundreds of different combinations of PTSD symptoms that could meet the diagnostic criteria, which makes it unlikely to come across two entirely identical PTSD cases. Psychiatric comorbidity in patients with PTSD is probably the rule rather than the exception, and this is the main reason for the extremely low diagnostic validity (14).

The case of ‘anorexia nervosa’ is a typical paradigm of a worldwide scientific misconception, having serious diagnostic and therapeutic consequences for the patients. For many decades ‘anorexia nervosa’, as indicate the label, is perceived as a disorder related to ‘lack of orexis’ (from the Greek ‘orexis’ for ‘appetite’ and ‘anorexia’ for the ‘loss of appetite’), having until now serious problems with therapy. Truth is that ‘anorectic’ individuals do not loss appetite. In fact, they just restrict feeding by stirring food, separating the food to be eaten, counting bites, cutting food into geometric shapes, cutting food into small pieces, chewing for a long time, taking long breaks between bites, and eating extremely slowly. Patients have an intense ‘sense of mastery/sense of control over world’, leading to an ‘over-control’ over her body shape and weight. In this frame, patients develop controlling behavior on eating, resulting in weight loss and body shape preoccupations. Is anorexia nervosa a self-healed disease, starting at about 13 years old, and ending 10 or 15 years later? And what is happening in these patients after this period? Do they suffer from any disorder? Truth is that these patients will continue to live in the same obsessive/compulsive realm, but without the ‘anorectic’ behavior. So, we need to think about eating disorders (ED) from an obsessive-compulsive disorder (OCD) perspective. Whether ED is ever reclassified as OCD or a related disorder in the DSM/ICD awaits future research, but the evidence is hard to ignore. This aspect will inform medication selection and dosage, for targeting eating-related obsessive and compulsive symptoms, and encourage relevant research (15, 16).

To reduce confusion about indications of psychiatric drugs Neuroscientists suggested a Neuroscience-based Nomenclature, that takes more into account the pharmacological indications and less the initial clinical observations (17). Moreover, the U.S. NIMH has elaborated a new framework for mental health research, the Research Domain Criteria, as an alternative method of classification with a dimensional approach, simultaneously defined by observable behavior, as well as neurobiological measures (18). The RDoC matrix distinguishes six domains of functioning (negative valence systems, positive valence systems, cognitive systems, social processes, arousal and regulatory systems, and sensorimotor systems) with various subconstructs and eight units of analysis: genes, molecules, cells, circuits, physiology, behavior, self-report, and paradigms. A key aspect concerns attention to mind-body issues that can begin to reconcile and integrate separate research traditions (e.g., phenomenology, behaviorism, neurobiology, and genetics) into a coherent view of mental disorders supported by empirical research. (19, 20).

Also, the Hierarchical Taxonomy of Psychopathology model describes a hierarchy of continuous dimensions accounting for broader spectra, as well as symptom-level manifestations of psychopathology (21). HiTOP facilitates investigation of neurobiological mechanisms at multiple levels of the hierarchy. Its dimensions provide targets that should be more useful for clinical neuroscience than binary diagnostic categories comprising heterogeneous symptoms. The HiTOP’s hierarchical structure enables both lumping and splitting approaches to psychopathology. For example, for a problem involving ‘fear’, the HiTOP lumps fear together with other forms of internalizing, thus recognizing important commonalities among all internalizing problems, which may benefit from similar treatment, but it also splits the fear subfactor from the other subfactors of internalizing. In other words, any given problem on a lower-level dimension may benefit from multiple interventions, some of which are also effective for other problems in the same spectrum or superspectrum and some of which are specifically effective for that problem (22).

It is worth noting that in biomedicine area there are thousands of distinct disease entities and concepts, each of which are known by different and sometimes contradictory names. To address these problems, a community of disease resources worked together to create the Mondo Disease Ontology as an open, community-driven ontology that integrates key medical and biomedical terminologies and is iteratively and regularly updated. It supports disease data integration to improve diagnosis, treatment, and translational research (23). Ontologies are structured frameworks for representing knowledge by systematically defining concepts, categories, and their relationships. In mood disorders, for example, the lack of formalized semantic models contributes to diagnostic inconsistencies, fragmented data structures, and barriers to precision medicine. The Human Phenotype Ontology (HPO) is a promising framework for bridging psychiatric and medical phenotypes, and there is an ongoing effort to enhance HPO through curated psychiatric terms, refined definitions, and structured mappings of observed phenomena (24).

## How did we get here

The term ‘neurology’ was coined by the English physician Thomas Willis, following his study of the anatomy of the brain in the 1660s. In 1808, Johann Christian Reil, a German physician and philosopher, coined the term ‘Psychiatry’. However, the two disciplines had common origins. The seminal work Mental Pathology and Therapeutics by Wilhelm Griesinger in 1845 still has a place in the 21st century. For Griesinger, mental illness was essentially a disease of the brain, a fact reflected in the title of the journal Archives of Psychiatry and Neurology that he founded in 1867. Over the years, researchers have emphasized other etiological factors, such as genetic and environmental, leading to the separation of psychiatry from medicine, proposed by Silas Weir Mitchell as early as 1894. Thus, the ‘handling of madness’ passed from the medical profession to the professional group of psychiatrists of the time. The split became even more pronounced in the United States between the 1930s and 1970s, when psychoanalysis and psychotherapy largely took over psychiatry.

By the end of World War II, the global system of psychological science had been severely fragmented, to be replaced by one in which there were and still are local differences. If we look outside Western Europe and North America, the situation becomes even more extreme. Some examples of cultural imperialism include France in relation to Argentina, Britain in relation to India, the United States in relation to Turkey, and even Japan in relation to China. Although there are certain patterns of dependence, the largest exporter of psychology was and continues to be the United States. There would be no problem here if the knowledge imported were culture free. In many Third World countries there is often a lack of “fit” between psychology and the local culture. The local population may not even think in psychological terms, and this is the case with the more traditional sectors of South African society. The Chinese didn’t even have the word psychology and translated it as “heart-spirit-study.” In general, however, the psychological concepts of each language are not identical. This is also true for European languages ​​such as German, Spanish, and French. The problem is even more extreme when one looks at Asian or African languages (25).

Today, approaching psychology more than neuroscience, psychiatry is limited to human subjectivity and existence, interpreting labyrinthine meanings and creating a narrative about narratives. According to Thibaut (11): “This isolation has seriously damaged psychiatry and caused important recruitment and funding problems, as well as diminished value of careful diagnosis, therefore reducing psychiatry to a nonspecific psychological support, which contributes to increasing the stigma”. Modern psychiatry has reduced the value of diagnosis, which has reduced the status of psychiatry to the provision of ‘non-specific psychological support’. The limitation of neurology to the study of the nervous system and psychiatry to the social brain has caused significant side effects in the diagnosis and treatment of mental disorders. In the real world today, the diagnostic and therapeutic space of mental illnesses is full of invalid diagnoses and is overwhelmed by countless and uncontrolled ‘treatments’, carried out by countless and diverse ‘therapists’ (26) (**Box 1**).

Box 1 **Psychiatry in the real world. The current situation in the field of mental health and the consequences in treatment of the mentally ill.**

Current situation

- Lack of strategic management,
- Lack of or very low referral rate for psychiatric evaluation,
- Reluctance to refer for psychiatric evaluation by doctors of other specialties,
- Alienation of psychiatry from other medical specialties,
- Lack of primary mental health care,
- Inadequate evaluation of mental health services,
- Lack of integration of neuroscience into psychiatry,
- Inappropriate naming in the mental health field,
- Stigmatizing naming of mental illnesses,
- Problematic diagnostic system - Lack of diagnostic validity,
- Lack of information about the therapeutic capacity of mental disorders by psychiatry,
- Distorted beliefs about the therapeutic capacity of medical versus non-medical specialties,
- Lack of certification of competence by non-medical specialties

Consequences

- Countless therapists with countless non-medical treatment techniques,
- The patient does not know where to address for the mental health problem,
- Few of them know what they suffer from,
- Most patients seek treatment in non-medical services,
- Few patients receive psychiatric treatment and follow-up,
- Lack of measurements of the flow of cases,
- Inadequate treatment of primary mental problems,
- Chronicity of mental problems,
- Accumulation of untreated cases,
- Increased risk of suicide,
- Increased risk of violent incidents and domestic violence,
- Increase in forced psychiatric hospitalizations,
- Poor treatment of co-morbid physical problems,
- Exploitation of mentally ill people,
- Burden on the health system
- Failures of prevention programs

Neurology, on the other hand, is based on fundamental neuroanatomical and neurophysiological relationships. Reinforced by the Cartesian mindset, the separation of neurology and psychiatry has led to a separation of ‘brain’ from ‘mind’, ‘physical’ from ‘mental’, which are useful elements for both disciplines. However, overlap is also evident in the relevant medical journals of the disciplines. A study of papers published in Neurology and the American Journal of Psychiatry found that less than 5% of the papers in the American Journal of Psychiatry dealt with meningitis, epilepsy and headaches, and less than 5% of the papers in Neurology dealt with schizophrenia, panic and mania. However, there is significant overlap in other categories. The proportions for ADHD were 23% and 77%, for autism 30% and 70%, and for mental retardation 70% and 30%, in Neurology and the American Journal of Psychiatry, respectively (27). Neurological diseases often manifest neuropsychiatric symptoms such as depression, anger, apathy and psychotic symptoms such as hallucinations and delusions. It should be noted that some neurological diseases are associated with 15% of acute and 70% of chronic psychiatric patients (28).

However, the increasing sophistication of etiologic psychiatric research has been accompanied by a greater focus on the biological and genetic causes of psychiatric disorders. The advent of diagnostic imaging and neuroscience has brought a high degree of clarity to the field. Kandel (29) even points out that if neuroimaging had been available in 1895, when Freud wrote the Project for Scientific Psychology, it would probably have steered psychoanalysis in a very different direction. All of this constitutes a major scientific paradox: Psychiatry, the science that for many decades has served significantly in understanding the functioning of the brain and mind, has lost its own intentional object (30, 31). Being outside the medico-social framework of operation, psychiatry now presents significant difficulty in ‘reading’ the basic properties and needs of its own scientific field. Aiming at integrated medico-social care, psychiatry must return to its ‘home’, that is, to the field of medicine, while leaving non-medical interventions to non-medical professionals (11).

## Need for change

In the real world, many psychiatric patients avoid appropriate treatment, ultimately resorting to non-medical treatments, sometimes with disastrous results. In addition to their unknown effects or side effects, these treatments are not approved for standard pharmacotherapeutic or psychotherapeutic rehabilitation interventions. Moreover, they may cause a delay in the implementation of appropriate treatment, sometimes even for a long time, which will worsen the mental illness and increase the likelihood of adverse or catastrophic events. The distorted naming of disorders, treatments, institutions, and specialties favors this general confusion. Moreover, fragmentation between physical and behavioral healthcare exacerbates the problem. Bearing in mind that physical health is a large determinant of mental health and vice versa, we note three key issues that need to be addressed: accessibility, quality, and cohesion of the mental health providing system. The ‘staging model’ offers a potential workable compromise between the dimensional and diagnostic approaches, recognizing opportunities for intervention at all stages of a disorder (32). ‘Timing’, also, is an important conceptual, research and therapeutic target for mental illnesses (33), together with the need to prioritize treatments for mental illnesses, in accordance with the valid discoveries of neuroscience (34). In parallel, to improve the effectiveness and efficiency of mental health services, a move from patient-centered care to patient-perspective care is recommended. An attitude of patient-perspective care would require service providers recognizing that help can only ever be defined by the helpee rather than the helper (35).

Psychiatric medication treatment is often associated with side effects and stigma. The ‘stigma’ exists because someone will go to a ‘psychiatrist’ and get medication for a ‘mental’ problem. However, it does not face such a problem when one goes to a neurologist for a neuropsychiatric illness, such as dementia or epilepsy, even though many neurological diseases are characterized by psychological symptoms. A good example is Autism, which over the years both the public and the scientific community have come to perceive as a developmental and therefore more as a neurological disease, resulting in both easier access for patients and their families to a doctor and the success of awareness campaigns (36). It is known that psychiatric stigma is deeply embedded in societal norms. It is a multifaceted issue permeating every level of psychiatric care, leading to delayed treatment, increased morbidity, and a diminished quality of life for patients. Addressing the stigma surrounding mental health can significantly enhance the effectiveness of psychiatric care (37). Having the experience of other stigmatized situations, such as HIV, we can design and deliver multiphase and multifaceted campaigns that included social marketing and mass media activity at the national level to raise awareness of mental health issues; Local community events to bring people with and without mental health problems together or a program to empower a network of people with experience of mental health problems to challenge discrimination; Also, targeted work with stakeholders, for example, medical students, teachers in training, employers, and young people (38).

The clinical manifestations of neurological diseases vary depending on the underlying pathology, affected anatomical structures, and disease stage. Symptoms may include cognitive impairment, sensory abnormalities and altered consciousness or behavior. Treatment strategies for neurological diseases aim to alleviate symptoms, slow disease progression, and improve patients’ quality of life. Pharmacological interventions are commonly used to manage symptoms, such as pain, seizures and mood disturbances. For example: anti-epileptic drugs are prescribed to prevent seizures in patients with epilepsy, Dopaminergic medications (e.g., levodopa) are used to alleviate motor symptoms in Parkinson’s disease and Immunomodulatory therapies (e.g., corticosteroids, disease-modifying drugs) are employed to reduce inflammation and disease activity in autoimmune disorders. In addition to medication, non-pharmacological approaches such as physical therapy, occupational therapy, speech therapy, and cognitive rehabilitation play a crucial role in managing neurological diseases (39).

Considering the similarities between neurodevelopmental disorders that are treated mainly by neurologists and those treated mainly by psychiatrists, we suggest it is unfair that serious psychiatric syndromes, such as individuals on the psychotic spectrum, do not receive the appropriate treatment. Schizophrenia is a complex, heterogeneous behavioural and cognitive syndrome that seems to originate from disruption of brain development caused by genetic or environmental factors, or both. Advances in genomics and neuroscience have led to great progress in understanding the disorder (40). Recent discoveries regarding inherent biomarkers and neurodevelopmental disease progression increasingly suggest possible overlaps with other psychiatric disorders (41). Today, schizophrenia is considered a typical psychiatric disorder, since it contains symptoms of a functional nature, and its treatment includes various psychological interventions. However, there are many indications of the existence of neurobiological causes even before the onset of the disorder. Also, pharmacological treatment is the main treatment and the prerequisite for the successful outcome of any non-pharmacological treatment session (42).

Social medicine analyses and describes the interactions between illness, individual, society, and organizational structures of the health care system, including prevention, treatment and rehabilitation. An ecosocial systems view offers a way for clinicians to organize the multiple explanatory models needed to capture the complexity and heterogeneity of psychiatric disorders and illness experience (43). A medico-social approach to the treatment of psychiatric disorders presupposes the basic involvement of professional psychiatrists, both in the first line of treatment and in subsequent referral processes. High psychiatric comorbidity often requires collaboration with subspecialties of psychiatry, such as addiction psychiatry, adolescent and child psychiatry, forensic psychiatry, geriatric psychiatry, etc. Also, high comorbidity with physical diseases requires collaboration and referral to other medical specialties, such as an endocrinologist, neurologist, pathologist, cardiologist, etc. Referral for special programs, psychotherapeutic treatment, psychoeducation or rehabilitation are often deemed necessary, to specialist non-medical health professionals, such as psychologists, social workers, etc. By keeping this system consistent, cases will have continuity of care (**Box 2**).

Box 2 **Need for change. Need for a central role of psychiatry in the mental health system and the collaborative referral system.**

With the psychiatrist at the center

- Promotion of a medico-social model of treatment,
- The psychiatrist must be present in the primary health care system,
- Primary psychiatric assessment of all individuals with mental health problems,
- Psychiatric diagnosis necessary in all mental health providing settings,
- Treatment planning in all mental health cases,
- Psychiatric therapy, where necessary,
- Psychoeducation, including the family members,
- Identifying naming problems in diagnoses and treatments,
- Reducing misconceptions,
- Strengthening the doctor-patient relationship,
- Appropriate referral to another medical specialty,
- Appropriate referral to a non-medical specialty,
- Effectiveness assessment

Collaborative referral system

- Utilization of subspecialties of Addiction Psychiatry, Adolescent and Child Psychiatry, Forensic Psychiatry, Geriatric Psychiatry, etc.
- Parallel treatment in psychotherapy, rehabilitation, or psychoeducation programs,
- Appropriate referral for specialized psychotherapy,
- Appropriate referral for a dietary program,
- Appropriate referral to a social worker for issues of occupational rehabilitation, retirement, disability benefits, etc.
- Appropriate referral to a doctor of another specialty, such as an endocrinologist, neurologist, pathologist, cardiologist, etc.,
- The psychiatrist must be the reference person for the entire therapeutic process of a mentally ill patient.

The use of primary care psychiatrists can increase access to primary care by persons with serious mental illness and improve outcomes (44). Maybe, psychiatrists need to be able to medically evaluate and to provide basic primary medical care for seriously mentally ill patients who do not have adequate access to general health care (45). The biopsychosocial approach championed by Engel (46) promised a conceptual framework to integrate multiple levels of analysis in psychiatry based on general systems theory. People exposed to more unfavorable social circumstances are more vulnerable to poor mental health over their life course. Groups who are often exposed to a multitude of intersecting social risk factors include refugees, asylum seekers and displaced per sons, as well as ethnoracial minoritized groups; lesbian, gay, bisexual, transgender and queer groups, and those living in poverty (47).

## Perspectives

In the history of the mental health journey, four trends have emerged in sequence: The first concerns “what” mental disorders are, from which George Engel’s biopsychosocial model emerged. The second answered the question “where” mental health is provided and involved the transition from “institutional care” to “community care”. The third concerned “who” is the provider of the service and the doctrine that ‘mental health is everybody’s business’. Mental health promotion, prevention, treatment of and recovery from mental disorders were no longer the prerogative of a single group of experts, a role historically played by psychiatrists. A diversity of persons have become active, from a range of mental health professionals to a range of non-specialist providers such as community health workers, teachers, as well as carers. Finally, the fourth shift is exemplified by the expression “nothing about us without us”, which is becoming a fundamental, rights-based component of the ethos of mental health care provision and research, which places the needs of persons affected at the heart of mental health care (40).

After so many years of experience and knowledge, we must accept that the issue of mental health is not, and should not be, an ideological issue. There is no need for more prejudices and delays. Obviously, any strategy in the future will have to combine in a harmonious way all the above trends, having as its sole objective the improvement of the mental health and wellbeing of the mentally ill. Until now, the often inappropriate naming of diagnoses and treatments, the low validity of diagnoses, the mediocre pharmaceutical and psychotherapeutic effectiveness, and the low utilization of neuroscientific knowledge, seem to be a common finding. Also, one of the greatest lessons we have learned is that the failure, to a greater or lesser extent, of all treatment and prevention programs to date is related to poor coordination and poor management of the flow of cases. As analyzed at length above, this dystopian flow begins from the first day the patient finds themselves in need of seeking a specialist, and, if they are not lucky, will continue for the rest of their life. If this “therapeutic discontinuity” is not addressed, any funding for staffing, salaries, infrastructure, psychosocial programs, etc. will steadily disappear, with minimal benefits for the patient. Similarly, valuable neuroscientific knowledge will only be utilized occasionally and in the way that anyone knows it. Based on the above, **Box 3** summarizes some recommendations for short-term and long-term action planning.

Box 3 **Short- and long-term recommendations for the useful functioning of mental health services.**

Sort-term recommended changes

Realistic treatment

A medico-social therapeutic model should be adopted in the field of mental health.

Using a common language of understanding, as much as possible, in areas of diagnosis and treatment.

Neuroscientific knowledge is meaningful when utilized in the field of mental health.

The psychiatrist is the key mental health professional who can connect neuroscientific knowledge with the needs of patients.

Any increase in mental health funding will only be effective if the flow of cases is managed by the psychiatrist.

Encourage novel understandings from diverse disciplines such as genomics, neuroscience, health services research, clinical sciences and social sciences.

Application

The flow of mental health cases should be based on psychiatric assessment.

Treatment planning should be an important part of the psychiatrist's work.

Adaptation to the respective arrangements of the diagnostic criteria.

It is necessary to choose the best possible treatments.

It is necessary a congentive treatment of mentally ill patients.

It is necessary to ensure the consistency of the various therapeutic interventions that are applied.

It is necessary to refer for psychiatric assessment of individuals with mental burden identified in the broader health field.

Tactics that delay or hinder the implementation of approved treatments should be identified and prevented.

Community collaboration

Public awareness on mental health issues.

Discussion with mental health specialists on the need of changing therapy culture, in conferences, meetings, articles, etc.

Investments for mental health must be substantially enhanced.

Improving access to psychosocial interventions.

Redistribution of mental health budgets.

Technological solutions and the use of digital technologies must be embraced.

Long-term recommended changes

Change of therapeutic mindset

Mental health services must be integrated into the global response to other health priorities.

Strategic planning with a focus on the medico-social care of mental health patients.

Countries have to utilise available planning tools to set their own targets for inputs (such as budgets, staff and beds), processes (such as numbers of skilled providers) and outcomes (such as improved mental health).

Any financial support will not be effective if the rules of ‘continuity’ and ‘coherence’ of treatment are not applied.

Applications

Strengthening the position of the psychiatrist in matters of therapeutic planning.

Linking neuroscientific knowledge and the treatment of mentally ill patients.

Constant relevant discussion and information of employees with articles, scientific events and staff meetings.

Actions targeted in developmentally sensitive periods early in the life course.

Innovative use of trained non specialist human resources and digital technologies to deliver a range of mental health interventions.

Monitoring and accountability for global mental health must be strengthened.

Fifteen years ago, Reynolds et al. (48) suggested the future of psychiatry as clinical neuroscience, noting: “Psychiatry is grounded in clinical neuroscience. Its core mission, now and in the future, is best served within this context because advances in assessment, treatment, and prevention of brain disorders are likely to originate from studies of etiology and pathophysiology based in clinical and translational neuroscience”. However, psychiatry continues to be divided into many sectors, trying to meet the multiple and unmet needs of the mentally ill. There are serious problems in the “continuity” and “coherence” of treatments, resulting in the continuous dispersion and leakage of resources. Each sector and subspecialty of psychiatry demonstrate an important scientific work, which in fact has excellent development prospects. Unfortunately, however, a small part of this work will continue to ultimately end up with the patient. A different management strategy is required, which is described in this article. If we explore other medical specialties, we will see that they have a much clearer landscape in the application of knowledge in recording results, but also in future perspectives.

In Oncology, for example, one-size-fits-all approaches to the treatment of cancer have been superseded by precision medicines that target specific disease characteristics, promising maximum clinical efficacy, minimal safety concerns, and reduced economic burden (49). In clinical endocrinology AI holds the promise to dramatically improve the way endocrinologists screen, diagnose, treat, monitor, and coach patients (50). In neurocardiology, cardiologists and neurologists now work closely in teams to better understand and treat conditions such as stroke and dementia and other conditions that involve heart and brain considerations (51). Finally, for the future of Neurology, the European Academy of Neurology, recently reported (52): “General Neurologists are essential to ensure rapid, accessible, and comprehensive (that is interdisciplinary and coordinated) management of patients with neurological disorders. They will remain essential for the appropriate management of patients in the communities where they live, providing coordinated and shared care with general practitioners (and other health professionals) on one side, and with specialized and hospital-based neurologists on the other. By doing this, General Neurologists can prevent an unnecessary (and expensive) fragmentation of care, which could result in patient harm, and the disintegration of neurology, as has already happened to general/internal medicine”.

Finalizing, psychiatric and neurological disorders represent a significant burden on global health (53). The paradigm of One Health Care, an integrated approach encompassing human, animal, and environmental health (54), has gained significant traction. It contains three distinct but interrelated dimensions: the shared environment, the safe food and food systems, and the shared medicines and interventions. A systematic scoping review showed trends included a predominate focus on companion animals as interventions, “sense of place” used as a component of mental well-being, and non-physical health-related measurements of animal well-being as an outcome within One Health research (55). While traditionally applied to infectious diseases and zoonoses, its principles are now extending to encompass psychiatric and neurological disorders (56). Clinicians and policymakers can collectively address the complex challenges of psychiatric and neurological disorders, promoting resilience, equity, and well-being across diverse populations (57). Finally, discussing the future of mental health epidemiology, Abdalla and Galea (58) considered a need for a renewed focus on the macrosocial determinants of mental health, proposing a more deliberate assessment of the mechanisms leading to adverse mental health outcomes, which can then be used to inform novel interventions.

## Conclusions

Society needs real words with clear meanings and not stigmatized. We need actual words in communication between patients, psychiatrists, clinicians, institutions, journals, pharmaceutical companies, legal agencies, and media. What really needs to be changed is the way that mental illness is seen by the public, while any such change will need to include assessments with patients and carers. The processes of renaming, redefining and reconceptualization of mental health are long and challenging, although there is no serious difficulty, beyond our own internal resistance to change (7, 26). Mental health professionals (psychiatrists, psychologists, social workers, etc.) will need to discuss the core weaknesses in service provision. They will need to understand the “discontinuity of treatment” and the “lack of coherence” in the treatment of mentally ill patients that occurs today. These phenomena tend to eliminate any financial support from the state, since the patients relapse and start over from scratch. Discussions on this topic can take place at all levels, such as at the level of a group, organization, policy makers, through specialized meetings, conferences, articles, etc.

Today’s diagnostic criteria reflect the outcome of different schools of thought about how to approach psychiatric diagnoses, sometimes referred to as splitting or lumping side of the argument. Lumpers tend to think of mental illness diagnoses as a spectrum, while splitters advocate separating diagnoses into multiple different subtypes on the basis that each one is in some way unique. In many medical conditions, a splitter approach is useful, but this doesn’t work in mental health, as it ignores the reality of patient experiences, and the fact that most individuals have mixed symptoms, due to comorbid conditions. McGorry et al. (59) outlined the candidate strategies of RDoC and HiTOP that could pave the way for such a paradigm shift, but they suggested that none is yet fit for purpose. However, the authors suggested clinical staging as a factor that aims to both expand the scope and refine the utility of diagnosis by the inclusion of the dimension of timing.

Society needs clear answers to resolve mental health issues. In this call, psychiatrists should find their role within medicine, having a central position in the field of mental health. They should update and apply the discoveries of neuroscience to improve the health and quality of life of patients. They should take on a central regulatory role in the flow of cases, referring appropriately to a clinician or to a non-physician mental health professional. Also, they should design and supervise the necessary psychoeducation and rehabilitation programs, guiding the team of non-physician mental health professionals, with the goal of improving patients’ functioning.

Finally, psychiatrists must be present in the current events of the planet, designing programs to identify, prevent or address harm, in the areas of climate change (60, 61), wars (62) and migration (63). In all of this, they now have digital technology and artificial intelligence as their allies (64, 65). On the other hand, the stakeholders involved should promote the view of a medico-social approach to mental disorders, centered on formal diagnosis and treatment by professionals, with the aim of providing safe, continuous and complete care for patients (10, 26).

## Data Availability

The raw data supporting the conclusions of this article will be made available by the authors, without undue reservation.
